# How Can We Size Your Core Issue? Assessing Salience Validity Using Psychophysiology

**DOI:** 10.1093/poq/nfaf041

**Published:** 2025-12-05

**Authors:** Camille Tremblay-Antoine, Yannick Dufresne, François Vachon

**Affiliations:** PhD Candidate, Political Science Department, Université Laval, Québec (QC), Canada; Associate Professor, Department of Political Science, Université Laval, Québec, QC, Canada; Professor, Department of Psychology, Université Laval, Québec, QC, Canada

## Abstract

Much research in public opinion attempts to operationalize and measure individual issue salience. Measuring this concept presents its own set of challenges, due in part to the fact that studies rely mostly on so-called “subjective” methods to measure the strength of attitudes. This paper aims to transcend the classical methods used in surveys to measure salience by comparing the results of these common approaches with results obtained with physiological measures. Using the Confirmatory Factor Analysis Model with the Correlated Uniquenesses method, correlations between three survey question methods and two physiological measurements are compared to measure individual issue salience. Results show a strong correlation between all the measures tested and therefore add validation to survey approaches used in social sciences to measure issue salience. The results therefore demonstrate that individuals know which issues trigger the most reactions in them.

## Introduction

Issue salience is a widely employed term in political science and communication. However, scholars have used the term “salience” imprecisely and inconsistently. Most frequently, scholars intend *salience* to mean the *subjective importance* of an issue to a citizen. Another common usage equates “salience” with the *accessibility of attitude* objects related to an issue. Others suggest that salience necessarily implies emotional intensity, without necessarily equating the two terms (i.e., one might cause the other or they may have a common cause). Usually, scholars envision salience to be a distinct concept, but Krosnick and colleagues (1993) speculated that salience might be one of several attitude attributes (such as centrality) that might be part of a single latent construct.

This sets up the paper’s key puzzle: if importance, accessibility, and emotional intensity are distinct, then the terms cannot be used interchangeably, conclusions about importance cannot be uncritically extended to accessibility and emotional intensity, and we should develop separate measures of each that demonstrate discriminant validity. But if these three concepts can be shown to be highly correlated with one another, then they are to some extent substitutable for each other: a measure of any one can be considered as a valid empirical indicator of a broad concept that we refer to as salience. This puzzle remains unresolved.

This paper adds additional evidence that is relevant to this puzzle. Using a set of fifteen societal issues (each part of one of five broader issue areas) and data from a nonprobability sample of 116 French-speaking Canadians, the paper reports on the intercorrelations among five alternative measures of salience. Three measures are based on a questionnaire: these include two measures of self-reported importance, plus a measure of accessibility derived from timing data when answering the importance questions. Two measures are based on a controlled lab experiment where subjects are exposed to words associated with each of the five issue areas. When responding to these words, pupil dilation and galvanic skin response were measured to generate two objective measures of emotional arousal.

## Issue Salience as Importance

In 2019, Dennison summarized the main studies in political science analyzing issue salience as having either a focus on policy attitude importance (Krosnick 1990), issue importance (Fournier et al. 2003), policy issue salience (Page and Shapiro 1983; Miller et al. 2016) and, even on broader concepts like importance, centrality, ego involvement, preponderance, or just salience (Krosnick et al. 1993; Oppermann and Viehrig 2009). He also observes that the definitions overlap, are fuzzy, or vary from one study to the other. This observation is not exclusive to the field of political science. In the realm of marketing and consumer analysis, conceptual and definitional ambiguity have also been noticed: “Salience and importance appear to have been used almost interchangeably in many studies involving attitude measurement and/or product positioning” (Myers and Alpert 1977, 106). Furthermore, in this field, salience is sometimes defined as the elicitation order of product or service attributes that are considered “important” (Myers and Alpert 1977; Morris 2013).

Researchers have tested methods that make it possible to avoid using only self-reported responses to measure salience while still being able to deploy large-sample estimates. The only objective salience measurement used in public opinion polls is response time to questions about an issue.

## Issue Salience as Accessibility

Accessibility, in the context of issue salience, refers to the ease with which information regarding an issue can be retrieved from memory and brought to consciousness. This concept is closely linked to the notion of cognitive availability, which posits that the more accessible an issue is, the more likely it is to influence an individual’s attitudes and behaviors (Zaller 1992). Accessibility can manifest in various ways, including the speed of recall during surveys (Bishop et al. 2009; Reisenzein and Franikowski 2022), the frequency of issue mention in discourse (Cohen 2015), and the immediacy with which an issue can be activated in decision-making processes (Mostafa and Kasamani 2020).

Research has shown that accessibility is a critical factor in determining how individuals prioritize issues in their political and social environments. For instance, Krosnick (1990) demonstrated that individuals are more likely to express strong preferences for issues that are top-of-mind at the time of decision-making. This phenomenon is reflected in how issues become salient during electoral cycles, where media coverage and public discourse can significantly enhance the accessibility of certain topics, thereby shaping public opinion (Iyengar and Kinder 1987).

A common method to assess accessibility involves measuring response times in surveys or experiments. The premise is that quicker responses indicate higher accessibility of information related to the issue at hand (Bishop et al. 2009; Reisenzein and Franikowski 2022). For instance, if respondents can rapidly recall their views on a particular issue, it suggests that the issue is salient in their cognitive framework (Gomez and Wilson 2001). Conversely, slower response times may indicate a lower level of accessibility, suggesting that the issue is less significant or less integrated into the individual’s belief system.

Furthermore, accessibility can be influenced by various external factors, such as recent media exposure or personal experiences. The agenda-setting theory posits that media plays a crucial role in enhancing the accessibility of specific issues by framing them in ways that capture public attention (McCombs and Shaw 1972). This can lead to increased public discourse, which reinforces the accessibility of those issues in individuals’ minds.

However, the relationship between accessibility and emotional intensity must also be considered. While an issue might be cognitively accessible, it may not necessarily evoke strong emotional responses. Research suggests that emotional intensity can enhance cognitive accessibility, making issues that provoke strong feelings more readily available in memory (Schwarz and Clore 2003). This interaction implies that high emotional arousal can increase the likelihood of quick recall, thereby linking the concepts of emotional intensity and accessibility.

## Issue Salience as Emotional Arousal

Variations in the level of salience, both emotional and cognitive, are based on the approach and avoidance theory of Mehrabian and Russell (1974). This theory relies on the fact that issues that greatly attract or repel an individual will generate a greater physiological reaction. Thus, the most salient individual issues would be those to which citizens strongly react in terms of strongly positive or negative interest and emotions (Elliot and Covington 2001; Eder, Elliot, and Harmon-Jones 2013). This same theory is used and similar results are confirmed in the field of consumer behavior research, where there is a strong correlation in the strength of salience between issues strongly repelling and attracting consumers (Kenhove and Desrumaux 1997; Foxall and Greenley 1999).

As in public opinion polls, the behavioral measure of the time to respond to a question on importance is also used in psychology and is associated with a *Simple Reaction Time Task*: the shorter the response time, the more important the probed issue is for the respondent who would more quickly retrieve the information in long-term memory to transfer it to their working memory for use (Bishop, Karageorghis, and Kinrade 2009; Reisenzein and Franikowski 2022). But most studies analyzing this emotional psychological reaction to issues rely on self-reported subjective answers to questions like “*How important is the following issue to you?*” or “*How do you feel about this issue?”* without, to our knowledge, the congruence between the answers to this type of question and the physiological reactions of individuals having been tested. Thus, in the cognitive and behavioral studies of issue salience, the main postulate in the causal mechanism that accounts for the effects of salience on thoughts and actions is that when individuals assign importance to an issue, it can activate and engage their emotional systems (Miller et al. 2016).

The salience of an issue also elicits an immediate emotional response linked to the activation of that issue in memory (Matthews et al. 2000). However, measuring the response time in an opinion poll does not make it possible to account for this reaction. Until now, it has been mostly the field of psychology that analyzes affective reactions with objective measures.

Skin conduction, or galvanic skin response (GSR), is a measure of the peripheral nervous system and is considered one of the best indicators of emotional intensity (Boucsein 2012; Lang, Potter, and Bolls 2009). In emotions theory, James’s central work (1863) suggests that physiological changes are temporally preceded by emotions. Peripheral responses, such as GSR, could act as cues based on which emotions are formed (Christopoulos, Uy, and Yap 2019, 395). This measurement can accurately detect certain changes in an individual’s autonomic nervous system when exposed to specific content. Thus, the greater the amplitude of the GSR, the more emotionally activated the individual is, so the more salient the issue is in the mind of the individual (Bishop et al. 2009; Reisenzein and Franikowski 2022).

Pupil dilation is also an indicator of emotional intensity. The pupil diameter can vary from 2 mm to 8 mm and is controlled by the antagonistic muscles of the iris, innervated by the central nervous system (Beatty and Lucero-Wagoner 2000; Marois and Vachon 2018). Brain stimulation causes activation of antagonistic muscles that retract the iris. The iris can also retract or expand thanks to a group of muscles called the pupillary sphincters, which are linked to the peripheral nervous system (Marois and Vachon 2018). The scientific literature does not seem to recommend the use of this measure to produce a diagnosis, but rather to provide observations on the overall changes individuals go through in a situation of emotional reaction and, in particular, as an index to discriminate against weak stimuli (Lang, Potter, and Bolls 2009; van der Wel and van Steenbergen 2018; Klackl and Jonas 2019).

## Are Different Aspects of Salience Aligned?

The diverse conceptualizations of salience—importance, accessibility, and emotional arousal—raise a critical question: Are these aspects aligned, or do they operate independently? Some conceptualizations of the broad concept of salience are beginning to be more consensual, such as that of Dennison (2019, 437), who separates salience studies into two categories: “those that have defined issue salience in psychological terms of how important an individual believes an issue to be (or how much thought they give to it, or similar) and those that defined salience in purely behavioral terms as the weight an individual gives to an issue when making behavioral choices.” Individual issue salience can be viewed as a concept in the “attitude triad” (affect → cognition → conation), or otherwise formulated as “how we feel, what we think and what we are inclined to do as a result” (Rosenberg et al. 1960). Dennison’s (2019) categorization takes root in the attitude triad, without, however, separating the cognitive aspect from the affect or the behavior. Understanding these interrelations is essential for advancing both theoretical and empirical discussions.

### Conceptual Overlap and Divergence

Previous literature suggests that different aspects of salience are often conflated. For instance, Krosnick et al. (1993) highlight that salience might overlap with other attitude attributes such as centrality and ego involvement, indicating that these constructs may be part of a broader, latent framework. This ambiguity can lead to confusion, as researchers might interchangeably use salience to refer to importance, accessibility, or emotional intensity without recognizing the distinct implications of each concept.

**Figure 1.**
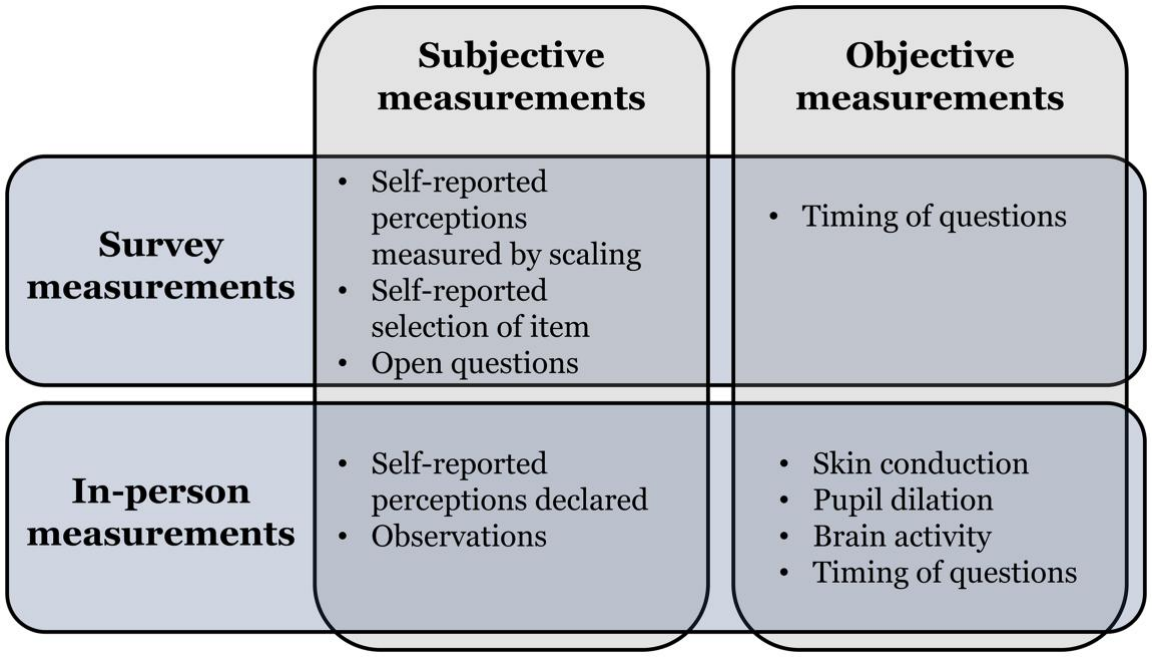
Typology of measurement types.

Furthermore, the measures used to assess salience (summarized in figure 1) can vary widely, contributing to divergent findings. Subjective measures—like self-reported importance ratings—are prone to biases stemming from social desirability and conscious psychological activity (Gawronski et al. 2015). In contrast, objective measures, such as physiological responses, capture unconscious processes that might influence individual salience perceptions. This distinction is crucial; if these measures yield inconsistent results, it undermines the validity of conclusions drawn from any single measure.

### The Role of Emotional Arousal

Emotional arousal plays a pivotal role in linking cognitive assessments of salience to behavioral outcomes. Theories of approach and avoidance (Mehrabian and Russell 1974) suggest that issues eliciting strong emotional reactions will generate pronounced physiological responses. Research indicates that high emotional intensity can enhance cognitive accessibility, thereby making certain issues more readily available for recall and decision-making (Schwarz and Clore 2003).

The interplay between emotional arousal and cognitive evaluations further complicates the salience landscape. Studies have shown that emotional responses can significantly influence how individuals prioritize issues in their political and social environments (Miller et al. 2016). However, while emotional intensity can heighten accessibility, the relationship is not straightforward; an issue may be accessible without necessarily provoking a strong emotional reaction. This nuanced understanding is essential for interpreting the results of studies examining salience.

### Bridging Objective and Subjective Measures

To address these complexities, our study employs both objective physiological measures and subjective survey responses in a single participant group. By comparing these different measurement approaches, we aim to clarify whether they assess the same underlying construct of individual issue salience. This leads us to our first hypothesis:

*H1: Physiological measurements and survey measurements of issue salience are independent measures of the same underlying construct.*

To empirically test this hypothesis, we will utilize a multitrait–multimethod (MTMM) approach (Campbell and Fiske 1959). According to this framework, valid yet independent measurement procedures assessing the same psychological construct should yield highly correlated results. By analyzing data from both self-reported surveys and physiological indicators (such as galvanic skin response and pupil dilation), we can investigate whether different measurement methods converge on a common understanding of salience.

### Assessing the Strength of Salience

The interplay between cognitive, affective, and behavioral dimensions also necessitates an examination of the strength of salience across different measurement types. Boninger et al. (1995) argue that salience effects are observable primarily at high levels of salience. Thus, we propose a second hypothesis:

*H2: Physiological measurements and survey measurements of issue salience will produce similar assessments of the strength of salience.*

This hypothesis posits that individuals are likely aware of the issues that elicit their strongest reactions, which they would also rate as the most salient. To assess this, we will categorize issues into low- and high-salience groups based on both physiological and survey responses, maximizing the comparative analysis between the two measurement types.

The implications of aligning objective and subjective measures of salience are profound. In public opinion research, reliance on self-reported measures has long been standard practice, yet this approach may overlook important nuances. Our study seeks to bridge this gap by integrating objective physiological assessments, thereby providing a more comprehensive understanding of individual salience. By validating these measures against each other, we can enhance the robustness of public opinion research methods and ensure that conclusions drawn from salience assessments are grounded in a more nuanced understanding of how people perceive and react to issues.

## Method

### Participants

The salience measurements are deployed on a nonrandom sample. The population used to measure the concept is that of Quebec adults. Participants^1^ were contacted separately via a single email (see Appendix A for the recruitment email). The institutional mailing list used to recruit participants consisted of a total of 53,826 people, which are current students and staff. The participants were selected based on the order of their response to the email and their eligibility according to the selection criteria. The recruitment stopped when the number of participants reached our goal of 150. The sampling method is therefore nonrandom, and participants are all volunteers. Quebecers suffering from anxiety or stress were not eligible for the experimental component since their stress could bias physiological measurements; potential participants were questioned on this point, and their self-reported response is used to include or exclude them. All participants came to the facility and completed both the online survey and the lab experiment on the same day between May 11, 2022, and October 13, 2022. They were compensated with 20 CAD for their time.

The goal of this article is not to draw conclusions about the most salient issues for the Quebec population, but to compare the reliability of the measurements. Thus, the nonrandom sampling method does not affect the reliability of the inference that is made between the measures since the group of respondents is identical from one measure to another.

### Online Survey

The questionnaire is developed with questions used in academic studies in social sciences to measure salience.^2^ It included a total of 20 questions divided into two types of measures: four selection-type questions and two scaling-type questions, each with multiple items. At the beginning of the survey, the respondents were kindly asked: “Please answer the following questions.”

The four selection-type questions asked respondents to choose a single issue (among Health, Environment, Economy, Culture, and Immigration) that they considered most important, least important, most likely to influence their vote, and least likely to influence their vote. These four questions were each presented on separate screens. Respondents were required to answer each question before proceeding, and no back button was available. A timer was embedded in the survey to record response times for these selection questions, providing an objective behavioral measure of salience.

The scaling-type questions aimed to capture subjective evaluations of a broader set of issues. Respondents were asked to rate eight issues (randomly selected from a list of 15) in terms of both their personal concern and their perceived importance. These two rating tasks were presented on two separate screens. The eight issues shown to each respondent were randomly drawn, and their order was randomized within each screen. However, the same eight issues appeared in the same order across both rating tasks for each respondent, ensuring consistency across concern and importance measures. These questions used 11-point scales ranging from 0 to 10.

The randomization procedures applied both to the order of the selection questions (which were randomized across respondents) and to the items presented within the scaling questions, ensuring variation in presentation and minimizing order effects. Appendix B presents the full structure and labeling of the online survey.

### Experimental Design and Material

The experiment was conducted in a dimly lit room on a PC computer running an E-Prime 3.0 (Psychology Software Tools) program. The program was used to present the instructions, control the presentation of the visual stimuli, and record the participants’ responses. The two physiological signals were preprocessed by Tobii Pro Lab software (Tobii Technologies). Pupillary measures were collected binocularly at a 300-Hertz (Hz) sampling rate (i.e., one recording every 3.33 milliseconds [ms]) with a Tobii Spectrum eye-tracking system (Tobii Technologies). Skin conductance was measured using a wearable Shimmer3 GSR+ sensor (Shimmer), which measures skin conductivity between two disposable tab electrodes attached to the index and middle fingers of the left hand. Signals were acquired with a sampling rate of 120 Hz and 16-bit resolution per sample. GSR signals were filtered using the Tobii GSR filter implement in Tobii Pro Lab, which applied a median filter with a 500-ms moving window followed by a mean filter with a 1,000-ms moving window.

#### The word as a stimulus

In order to maximize control, written words are used as a stimulus. Words related to each of the five issues studied were selected using the *Lexicoder Topic Dictionary*, developped by Young and Soroka (2012). Words categorized as neutral were also included in the corpus in order to provide physiological responses that allow measurement of the stimuli of the reactions’ base levels. For the selection of neutral words, the database *Lexique 3*^3^ is used. Then, controls in terms of effects of the physical presentation of the words and effects of the words’ meanings are used.

On the physical presentation, the words’ number of letters, phonological uniqueness, and number of syllables are controlled. Pupil dilation is influenced by the difference in light. The work of Hess (1972), replicated by De Winter et al. (2021), demonstrates that pupil dilation is correlated with the luminance to which the eye is exposed as well as mental demand. The length of a word therefore influences the light captured by the pupil. The word length also influences the amount of brain activity required to read the word. The use of longer words could cause a lag in the reaction and a flattened signal by the processing of the information (De Winter et al. 2021). Thus, all the selected words contain between 5 and 14 letters (with standard deviation between 1.50 and 2.87 in groups). This similarity was further enhanced by the use of words with more or less the same letters (phonological uniqueness).

The meaning of a word can also influence respondents’ physiological reactions (Mohammad 2018; Osgood, Suci, and Tannenbaum 1957; Russell 2003). Many studies support the hypothesis that more exciting words produce higher pupil dilation than nonexciting words (De Winter et al. 2021; Janisse 1977). Three dimensions are considered in the meaning of words: their *valence* (their positive/negative connotation), their *arousal* (their active/passive character), and their *dominance* (their dominant/submissive nature). These three characteristics cause particular physiological reactions in individuals (Mohammad 2018). The words for each issue studied are balanced for these characteristics by using the *National Research Council Valence, Arousal, and Dominance Lexicon (NCR VDA Lexicon)* (see table C.1 for each characteristic by selected words).

Another characteristic used to control physiological reactions is the word frequency in books. Word frequency is a very important factor in word recognition (New et al. 2004). The frequency of the word according to the corpus of books used in *Lexique 3* was therefore used to select words so that the t-test between the five issues corpus has a similar score (between 0.132 and 0.202).

Finally, to ensure that words included in the final corpus are good representatives of the issues that aim to be measured, the lists of words for each issue were validated using *crowdsourcing*. This method was used by Mohammad (2018) to ensure a similar selection in the reactions to words. All the identified words (172 words that meet the selection criteria previously stated) were presented in a survey on the Amazon Mechnanical Turk crowdsourcing platform (n = 80). Respondents on this platform identified, on a scale of 1 to 10, the relevance of each word to the issue it is supposed to represent. We selected nine words per category to include in the experiment among words with the highest scores of relevance. Neutral words that were linked to an issue category were excluded. Table 1 shows that differences between neutral and issues’ groups are not significant (all *p*-values are *p *> 0.05).

**Table 1.** T-test results of word characteristics by category compared to neutral words.

Category	Valence	Arousal	Dominance	Book frequency	Number of letters	Phonological uniqueness	Number of syllables
Economy	0.387	0.296	0.355	0.192	0.243	0.500	0.422
Environment	0.402	0.094	0.486	0.201	0.426	0.390	0.160
Health	0.168	0.196	0.490	0.209	0.362	0.192	0.223
Immigration	0.412	0.411	0.477	0.132	0.381	0.353	0.122
Culture	0.166	0.194	0.499	0.142	0.171	0.402	0.340

*Note:* t-tests of the five groups of n = 9 compared to the neutral group of n = 9.

### Procedure

When the subject arrives, the nature of the study, the course of the project, the benefits and drawbacks of participation, as well as the confidentiality and data management terms are presented. The subject then gives informed consent to participate in the project. The participant is then guided to a dimly lit room where a computer and the measurement instruments are set up. The sensors for pupil dilation and skin conductance are then calibrated and installed.

Once the setup was complete, participants were randomly assigned to begin with either the online survey or the laboratory experiment. Both tasks were completed consecutively, in the same room, using the same computer. The survey and experiment were administered under the same environmental conditions.

**Figure 2.**
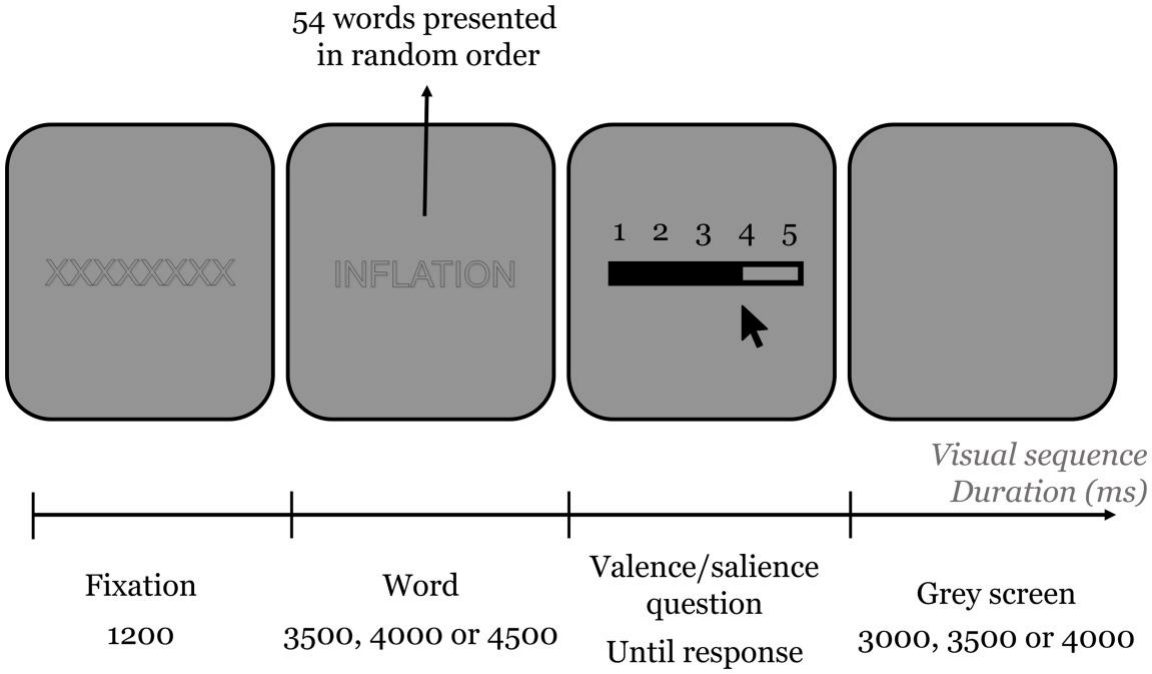
Trial sequence of the experiment. Schema inspired from Wardhani, Boehler, and Mathot (2022).

During the laboratory experiment, respondents are exposed randomly twice to each word of the corpus (total of 108 words). Figure 2 presents the visual sequence. Each trial begins with a fixation of 1,200 ms on 8 X’s. The stimulus causing the difference in luminance of the word on the image is reduced by using the method developed by Polt and Hess (1968), allowing us to diminish the difference in light while changing the projected words. Researchers have shown that using a gray background with an empty font (see figure 2) allows for reducing pupil dilation induced by changes in luminance (De Winter et al. 2021, 197).

“Jittering”—the introduction of some variability in the temporal distance between stimuli—is integrated in order to reduce the potential passivity of the participants (Christopoulos et al. 2019). Thus, the time a word is presented varies randomly for a duration chosen equiprobably between 3,500, 4,000, and 4,500 ms.

A screen then appears presenting randomly one of these two questions: (1) “To what extent does the word generate a pleasant or unpleasant feeling?” or (2) “To what extent does the word reflect something important to you?” The participant then evaluates the word according to the question on a scale of 1 to 5 by clicking on a number on the screen with the computer mouse. The question screen remains until the participant answers. These questions aim to keep participants attentive to the meanings of the words while keeping their eyes fixed on the screen. The answers to these questions are not used for this analysis.

The last screen of a trial is empty. Physiological reactions are recorded continuously throughout the duration of the experiment.

Encoding issues led to the exclusion of 18 participants from the study due to lack of data. For pupil dilation, participants for whom less than 75 percent of the signal has been captured during the experimental phase were excluded. Thus, 16 participants were excluded for this factor. Of the 150 participants, data from 116 were used for the analyses.

### Data Processing

For data from the physiological measurements and from the survey, several treatments were carried out to allow comparability between the participants as well as between the measurements.

**Figure 3.**
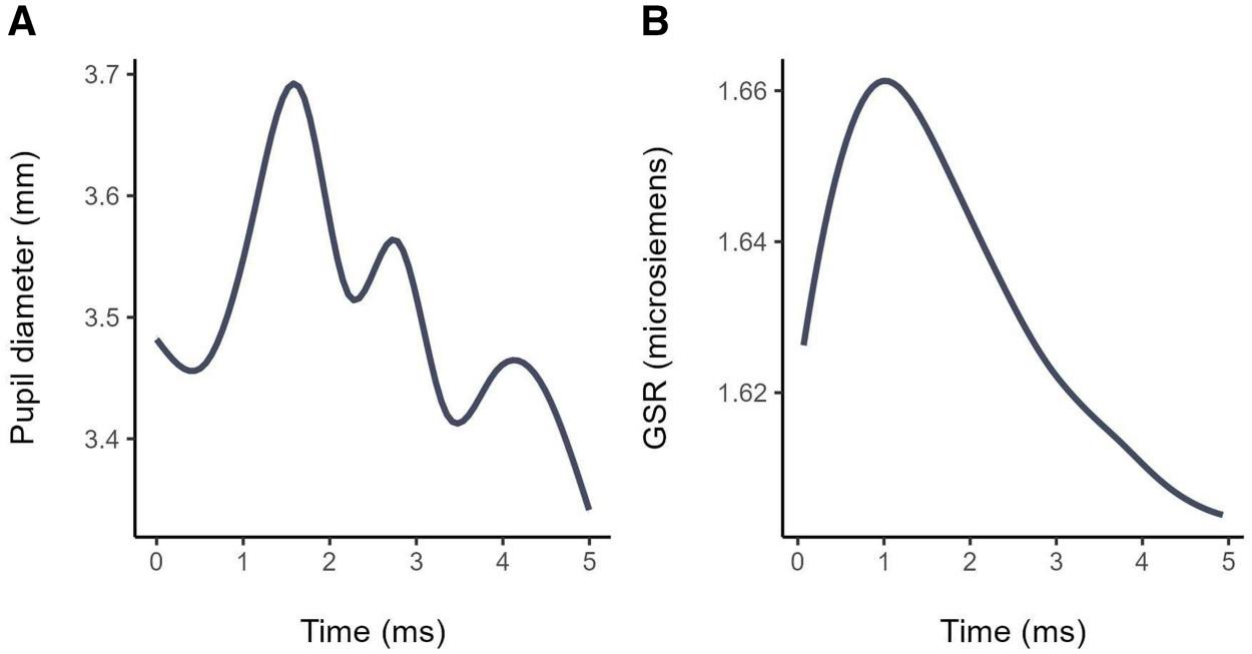
Dynamics of pupil and skin conductance responses. (A) Pupil diameter following the presentation of the stimuli. (B) Galvanic skin response following the presentation of the stimuli. This is an example of a recorded trial from respondent #2.

Dynamics of physiological responses are shown in figure 3. Typically, pupil size increases quickly at the presentation of the word. After exposure to the stimuli, the pupil diameter then decreases gradually. A similar response is recorded with the GSR. The rate of sweating increases in the first two seconds after the presentation of the word, then decreases. Removed data and missing data points originating from malfunctions or blinks were linearly interpolated using the pupil dilation data. Trials with 50 percent or more of interpolated data were rejected (908 trials rejected for the pupil dilation).

Data for all measurements were transformed by normalization to be on a scale of 0, low salience, to 1, high salience (this process is detailed in Appendix D).

For each type of measure, the issues were then ordered, from the one obtaining the lowest aggregated salience score to the one with the highest salience score.^4^ In 85.3 percent of the cases, all types of measurements identified the same issues as being the least salient for the same individual, and 89.7 percent for the most salient. For the remaining percentages, the issues integrated in the analysis as the least or most salient were identified based on the majority of measures identifying them as such. In all these cases, three or four measures identified the same issues; no participant had divergent results in terms of identification with more than two measures.

## Results

**Figure 4.**
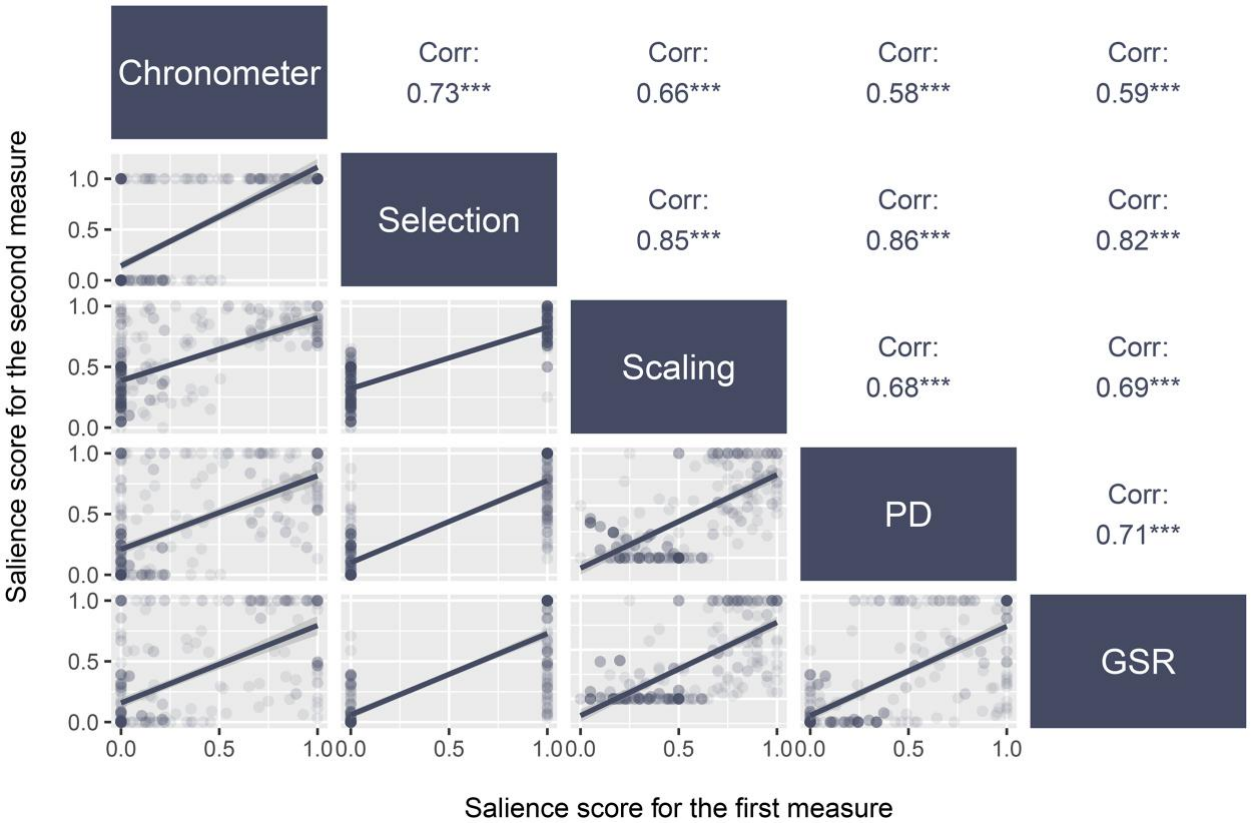
Salience index correlation matrix by measure. Correlation coefficients by pair of measurements and plotting of both measures by respondent with a linear regression line. Data from standardized coefficients. n = 116.

All analyses present results from unweighted data. Based on the multitrait–multimethod (MTMM) approach that establishes construct validity of psychological measures (Campbell and Fiske 1959), a correlation matrix was created to analyze, for the most and least salient issue by respondent, the relationship between each pair of measures. Figure 4 presents, in the lower left corner, the plotting of paired measures with a linear regression line (data distributions for all issues are presented by measure in Appendix E). We can see how the data is distributed on the two dimensions, which is in two groups for each pairs: one where the data approaches zero on both axes and the other where the data is near the maximum salience score of 1. This data modelization makes it possible to observe the combined distribution of participants’ lowest and highest salient issue by measurement, and the trends between each measure appear to be similar. These correlations between measures of the same construct assessed with different methods constitute primary evidence of convergent validity (Campbell and Fiske 1959). The correlation matrix is, on figure 4’s upper right, summarized with the correlation coefficients for each pair of measurements. With positive correlation coefficients and *p*-values all smaller than 0.001, validity coefficients are significantly different from zero. Thus, the matrix analysis demonstrates a strong correlation between measurements.

Furthermore, by modeling the proximity relationships between the measures by factor analysis, a common variance between all the measures is observed. In its initial use, confirmatory factor analysis allows testing validity of psychological measures by imposing theory-driven parameters in terms of factor loading, factor variance, and covariance, as well as error variance and covariance (Bong and Hong 2010). Survey measures are included here in the confirmatory factor analysis since, theoretically, they measure the same construct. Precisely, the Confirmatory Factor Analysis Model with Correlated Uniquenesses (CFA-CU), introduced by Marsh, Byrne, and Craven (1992), is used. This method has the advantage of eliminating method factors from the model and to specify correlated uniquenesses instead (Bong and Hong 2010). The factor analysis model indicates correlations among the uniqueness terms of the measures evaluated by each method distinctly. In CFA-CU, the model assumes that the variance in each measurement item that is not accounted for by the underlying latent trait (common factor) is attributed to method-specific or random error. The model can be mathematically represented as follows:


∑=ΛΘΛ+Ψ

where ∑ is a population correlation matrix, Λ is a matrix of factor loadings, Θ is a matrix of factor correlations, and Ψ is a diagonal matrix of uniqueness variances (Marsh, Byrne, and Craven 1992).

**Figure 5.**
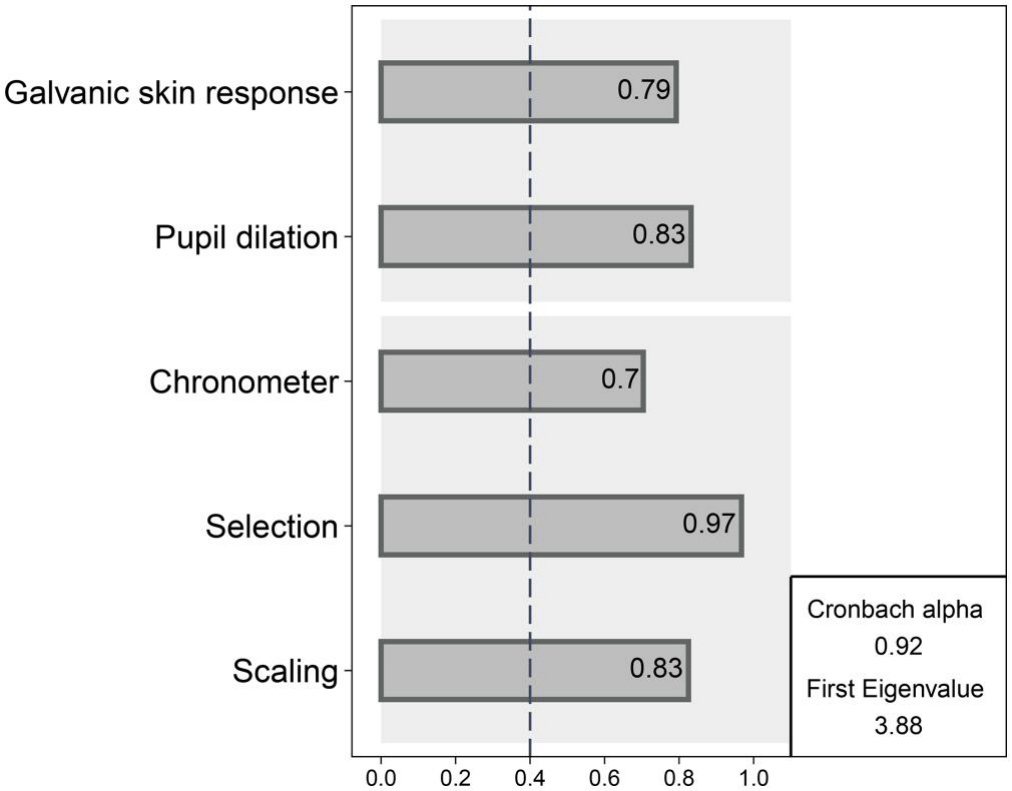
Reliability of each measurement to capture the same concept. Confirmatory Factor Analysis Model with Correlated Uniquenesses. Data from standardized coefficients. n = 116.

Figure 5 presents these factors’ loadings for each measure. For all measurements, the saturation coefficients exceed the commonly accepted threshold of 0.4 with a smallest coefficient of 0.7 for the response time measurement. The first eigenvalue for the correlation matrix is 3.88, which indicates a high variance of the construct that can be accounted for with a linear model by every single measure used. Finally, the internal consistency of the matrix measurements is estimated with the Cronbach alpha (*α*). The resulting *α* coefficient is 0.92, a score approaching 1, confirming that all of the measurements have high covariances.

Finally, we analyze which part of the issue salience can be explained with one or a combination of measurements. Table 2 presents the results of linear mixed-effects models of survey measurements, alone and aggregated, on physiological measurement score of issue salience.^5^ This approach also allows us to control for within-participant dependence in the linear regression models. For each of the three survey measures observed separately, the relationship with issue salience measured by physiological measure is strong. For all the survey measures integrated alone in a model (models 1, 3, and 5), the regression coefficients are all positive with a high statistical significance (*p *< 0.001), allowing us to conclude that there is a positive relationship between the score of each survey measure and the score established by physiological measures.

**Table 2.** Test of the explanatory nature of salience for each type of survey measurement.

Physiological measurement of issue salience								
	(1)	(2)	(3)	(4)	(5)	(6)	(7)	(8)
Chronometer	0.694	0.699					*−*0.058	*−*0.054
	*p *= 0.000	*p *= 0.000					*p *= 0.061	*p *= 0.084
	(0.042)	(0.042)					(0.031)	(0.031)
Selection			0.684	0.685			0.758	0.759
			*p *= 0.000	*p *= 0.000			*p *= 0.000	*p *= 0.000
			(0.013)	(0.013)			(0.032)	(0.032)
Scaling					1.111	1.125	*−*0.088	*−*0.096
					*p *= 0.000	*p *= 0.000	*p *= 0.114	*p *= 0.092
					(0.043)	(0.043)	(0.056)	(0.057)
Women		*−*0.028		*−*0.021		*−*0.011		*−*0.028
		*p *= 0.469		*p *= 0.427		*p *= 0.767		*p *= 0.301
		(0.039)		(0.027)		(0.039)		(0.027)
No university diploma		*−*0.149		*−*0.065		0.000		*−*0.063
		*p *= 0.044		*p *= 0.197		*p *= 1.000		*p *= 0.207
		(0.073)		(0.050)		(0.050)		(0.050)
Graduate diploma		*−*0.120		*−*0.089		*−*0.070		*−*0.086
		*p *= 0.031		*p *= 0.019		*p *= 0.200		*p *= 0.022
		(0.055)		(0.037)		(0.054)		(0.037)
Between 18 and 24 years old		*−*0.057		*−*0.031		*−*0.188		*−*0.021
		*p *= 0.227		*p *= 0.344		*p *= 0.025		*p *= 0.526
		(0.047)		(0.033)		(0.047)		(0.033)
45 years old and older		0.005		*−*0.038		*−*0.188		*−*0.025
		*p *= 0.9445		*p *= 0.423		*p *= 0.008		*p *= 0.597
		(0.071)		(0.047)		(0.069)		(0.047)
Constant	0.165	0.249	0.069	0.126	*−*0.247	*−*0.166	0.103	0.158
	*p *= 0.000	*p *= 0.000	*p *= 0.000	*p *= 0.000	*p *= 0.0000	*p *= 0.125	*p *= 0.000	*p *= 0.000
	(0.019)	(0.037)	(0.011)	(0.020)	(0.028)	(0.040)	(0.020)	(0.036)
N	232	232	232	232	232	232	232	232
Log likelihood	*−*44.535	*−*49.663	187.752	180.322	19.846	14.886	186.662	179.153
AIC	97.069	117.327	*−*367.503	*−*342.643	*−*31.691	*−*11.773	*−*361.325	*−*336.307
BIC	112.167	151.298	*−*352.405	*−*308.672	*−*16.593	22.198	*−*338.677	*−*295.099

*Note:* Linear mixed-effects models.

Data from standardized coefficients.

Standard errors presented in parentheses.

In model 7, the three survey measures were integrated into the same model to test whether a combination of the measures affects the link with the results drawn from the physiological reactions. Indeed, considered together, the three measures do not show the same relationship with the dependent variable. Only the selection measurement keeps a strong relationship with the physiological score of issue salience (with *p *< 0.001). However, the direction of the relationship for the timing and scaling measurement does not remain the same; the regression coefficient for both measurements becomes negative and close to zero (respectively, −0.06 and −0.09) and therefore loses its statistical significance (*p *= 0.061 and *p *= 0.114).

Sociodemographic characteristics of the participants are also added in separate models (models 2, 4, 6, and 8) to observe whether variations in the links between the measures may be due to respondents’ profile, and no effect on the relationship between survey and physiological measures is observed.

## Discussion

This paper’s goal was to assess whether the same results of individual issue salience are obtained when measuring the physiological reactions of individuals and when questioning them through surveys. Results obtained by pupil dilation and galvanic skin response on five political issues were compared with results from subjective survey questions measured by scaling and selection, but also with the objective survey measurement of response time. In the literature review conducted as a basis for this research, we identified 21 studies on the salience of issues that begin by listing the different conceptual and operational definitions of the concept and deploring the lack of consensus in the field. As research usually looks at the “attitude triad” of salience (affect *→* cognition *→* conation) by focusing on one aspect of salience, two at a time, or all of them without specifying, the results obtained in this research make it possible to be confident that this nevertheless measures the same underlying concept.

Since it is easier and less invasive to use self-report measures to capture public opinion salience (which is the most popular method in public opinion), the results of this paper show that there are no contraindications to do so. Furthermore, research results using self-report measures can be compared to research results using physiological measures. Overall, these research results should encourage better dialogue between social science disciplines studying the salience of public opinion with different methods.

When data obtained by isolated survey measurements is compared to that obtained by physiological measurements, the correlation between the strength of the salience score is strong and positive. This correlation persists even when controlling for participants’ sociodemographic variables. These results make it possible to draw conclusions on the validity of survey measurements of the scaling, selection, and chronometer type to measure the strength of the salience of an issue for an individual. The integration of the three survey measures in the same regression model shows that the selection measure (therefore asking the respondent himself to identify the issue he considers the most or least important) stands out among others. These results suggest that this measure should be prioritized in polls if a choice must be made among the three survey measurements when the aim is to identify the most or least salient issue. On the other hand, measures of response time and scaling can provide more information on how important an issue is for a person. They also provide more granularity in measuring the strength of salience.

It is worth mentioning some limitations of this study. First, this study was conducted on Canadians in a nonrepresentative sample. In future research, it would be relevant to test whether the robustness of survey measures to identify the salience of issues remains if the measure is exported elsewhere. Since the reliability of the measures remains when controlled with the sociodemographic groups during this experiment, one could suppose that similar results could be observed if the measure is generalized. And second, the research design of this study was based on the observations of Boninger et al. (1995) and Lavine et al. (1996), specifying that the effects of salience are observable only for high levels of salience. Thus, only the least and most salient issues for each individual are analyzed in this research. This methodological choice had the advantage of making it possible to integrate “selection” type questions into the questionnaire (where respondents can only select one issue). But overall, the results regarding the correlation of the salience strength obtained between all measurements allow us to be optimistic about the possibility to export the conclusions to moderately salient issues, but this remains to be confirmed in future studies.

More generally, this multidisciplinary project allowed us to identify practices used in psychology that studies in public opinion using surveys would benefit from using. When using words, a good practice in the psychology field is to use the *valence*, *arousal*, and *dominance* characteristics of words, since these affect the reactions of individuals (Mohammad 2018; Osgood, Suci, and Tannenbaum 1957; Russell 2003). These word data are available online and accessible to everyone. In the field of public opinion, it would be relevant to control the effect of words on the reactions of individuals. The lexicon used in this study, the *NCR VDA Lexicon*, allows us to know the words that cause the most reactions, which allows us to question the way in which question formulations can influence survey results.

Individuals do not care about the same issues (Miller and Peterson 2004; Dufresne and Ouellet 2021; Krosnick et al. 1993), and citizens’ positions on various issues can have a significant impact on their behaviors. If methods are more reliable in measuring positional issues—that is, nonconsensual issues whose positioning of an entity has the potential to change citizens’ behavior—it is possible to better understand society phenomena. Finally, regarding Krosnick et al.’s theoretical recommendation (1993) to consider issue salience as a single dimension for better theory building (see the introduction of this article), the results of our convergent validation lead us to encourage the public opinion community to move forward.

## Figures and Tables

**Figure E1.**
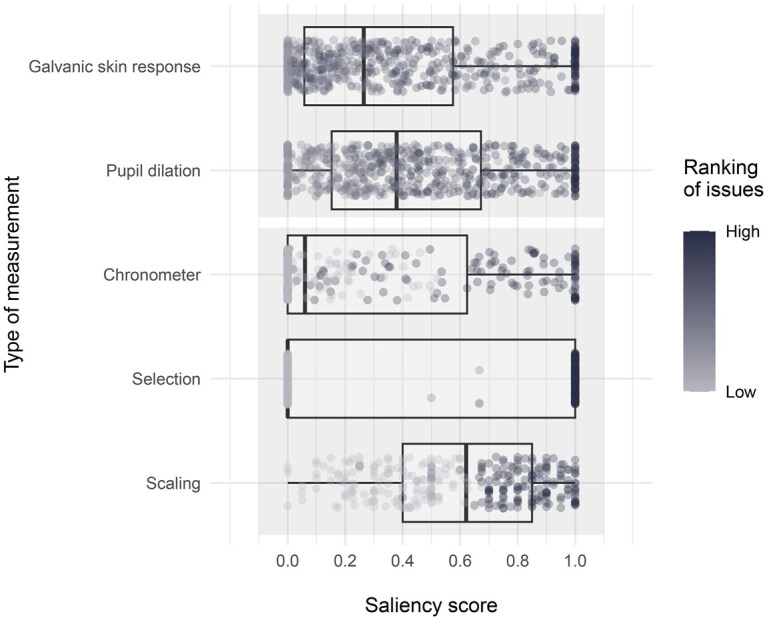
Salience scores of each issue per participant. Distribution of the saliency score by issue by type of measure. n = 116.

## Data Availability

The data underlying this article are available at https://osf.io/jv5z8/.

## Footnotes

1 This project was approved by the Ethics Committee of Université Laval (CÉRUL Approval No. 2021-309/12-11-2021). Participation was voluntary and carried out after informed consent.

2 A scoping review was carried out prior to this project. This systematic process made it possible to identify 176 studies measuring issue salience.

3 A computerized lexical database containing 35,746 lexical entries.

4 This method is used in order to compare all the measures with each other, as the selection measures only allow the identification of the most and least salient issue.

5 Linear mixed-effects models on PD and GSR are also presented separately in Appendix F for each survey measurement. No significant difference is found.

## Appendixes

### Appendix A. Translated Recruitment Email

#### Search for Participants

##### The salience of electoral promises: its tools, measurements, and validity

As part of a study aimed at creating a more valid measure of the salience of electoral promises, we are seeking Canadian citizens interested in completing a survey. To participate, you must:

- Be a Canadian citizen;
- Be of legal age;
- Not suffer from anxiety.

Your participation in this research consists of answering questions as part of an online questionnaire, which will take approximately 5 minutes, and coming to the laboratory of the School of Psychology at Laval University for an experiment lasting approximately 30 minutes. Both parts will cover the following elements:

- Information about you and your sociodemographic characteristics;
- Questions on the issues considered to be the most or least important;
- Exposure to words and physiological measurements (skin conductance and pupil dilation).

Participation is voluntary. If at any time you wish to withdraw from the survey or the laboratory experiment, you may do so. The completed questionnaire and the data collected in the laboratory will be kept confidential. Access to the study data will be restricted to the principal researcher.

You will receive an amount of $20 to compensate for any costs incurred by your participation in this research project.

This research project is part of Camille Tremblay-Antoine’s doctoral thesis, “The Salience of Electoral Promises: Its Tools, Measurements, and Validity,” supervised by Yannick Dufresne, associate professor in the Department of Political Science at Laval University. The project has been approved by the Laval University Ethics Committee (CÉRUL Approval No. 2021-309/12-11-2021).

For any questions, you can contact the following email address: camille.tremblay-antoine.1@ulaval.ca.

### Appendix B. Online Survey

The survey questions were translated into English but administered in French due to recruitment in Quebec, Canada’s French-speaking province.

For each question, the respondents had to make choices relative to the five issues (health, environment, economy, immigration, and culture). This selection of five issues to measure perceptions of importance is based on the conclusions of Ansolabehere, Rodden, and Snyder (2008), which have shown that it is necessary to prioritize the number of indicators per issue over the number of issues in order to analyze the strength of the salience. These five specific issues are selected because of their preponderance in the media coverage (Walgrave and Van Aelst 2006) and, therefore, of citizens’ knowledge of these issues (Soroka 2003).

**Table B1.** Survey questions and measures.

Measure	Question introduction	Items	Answer set
Selection; chronometer		Which of the following issues are of least importance to you?	Health, environment, economy, culture, immigration
Scaling	Please rate each of the following events in terms of your personal concern for them.	International trade tensions Increasing costs in the real estate market Rising taxes Air pollution increase Establishment of natural gas export projects Port expansion projects Shortage of healthcare professionals Long waiting times in the healthcare system Healthcare professionals under pressure Accessibility for all to cultural activities Support for the Canadian film industry Valuing Canadian heritage Welcoming refugees Integration of immigrants Delays in issuing permanent residence status	Not concerned 0, 1, 2, 3, 4, 5, 6, 7, 8, 9, 10 Very concerned
Scaling	How important are the following issues to you?	International trade tensions Increasing costs in the real estate market Rising taxes Air pollution increase Establishment of natural gas export projects Port expansion projects Shortage of healthcare professionals Long waiting times in the healthcare system Healthcare professionals under pressure Accessibility for all to cultural activities Support for the Canadian film industry Valuing Canadian heritage Welcoming refugees Integration of immigrants Delays in issuing permanent residence status	Not important at all 0, 1, 2, 3, 4, 5, 6, 7, 8, 9, 10 Very important
Selection		In an election, for which of the following issues is the positioning of political parties likely to influence your vote?	Health, environment, economy, culture, immigration
Selection; chronometer		In the context of the next Quebec provincial elections, what is the issue you consider: -the most important priority for you:	Health, environment, economy, culture, immigration
Selection; chronometer		In the context of the next Quebec provincial elections, what is the issue you consider: -the least important priority for you:	Health, environment, economy, culture, immigration

### Appendix C. Selected Words for the Experiment and Their Characteristics

**Table C1.** Selected words and their characteristics.

Category	Word	Translated word	Valence	Arousal	Dominance	Book frequency	Number of letters	Phonological uniqueness	Number of syllables	AMT mean
Economy	banque	bank	0.56	0.33	0.68	30.88	6.00	3.00	1.00	9.50
	budget	budget	0.64	0.40	0.73	5.34	6.00	5.00	2.00	9.70
	croissance	growth	0.92	0.59	0.78	3.99	10.00	7.00	2.00	9.20
	déficit	deficit	0.40	0.45	0.36	1.42	7.00	7.00	3.00	9.20
	dette	debt	0.20	0.60	0.34	12.16	5.00	3.00	1.00	9.30
	inflation	inflation	0.22	0.79	0.51	2.30	9.00	7.00	3.00	9.40
	investissement	investment	0.72	0.59	0.75	2.77	14.00	8.00	5.00	9.70
	marché	market	0.72	0.48	0.52	57.36	6.00	5.00	2.00	9.50
	revenu	income	0.75	0.51	0.728	2.23	6.00	6.00	3.00	9.20
Mean			0.57	0.53	0.60	13.16	7.67	5.67	2.44	9.41
Standard deviation			0.25	0.14	0.17	19.03	2.87	1.80	1.24	0.20
Environment	biodiversité	biodiversity	0.81	0.47	0.71	0.00	12.00	5.00	5.00	9.10
	climat	climat	0.64	0.24	0.48	17.16	6.00	5.00	2.00	10.00
	composte	compost	0.48	0.404	0.441	0.14	7.00	5.00	2.00	9.50
	écologie	ecology	0.89	0.29	0.61	0.20	8.00	7.00	4.00	9.90
	écosystème	ecosystem	0.73	0.26	0.63	0.00	10.00	5.00	4.00	9.90
	nature	nature	0.86	0.18	0.64	95.88	6.00	5.00	2.00	9.90
	ozone	ozone	0.56	0.35	0.52	0.54	5.00	3.00	2.00	9.10
	pollution	pollution	0.06	0.69	0.45	1.15	9.00	5.00	3.00	9.80
	préservation	preservation	0.667	0.51	0.686	0.47	12.00	9.00	4.00	8.60
Mean			0.63	0.38	0.57	12.84	8.33	5.44	3.11	9.53
Standard deviation			0.25	0.16	0.10	31.63	2.60	1.67	1.17	0.49
Health	chirurgie	surgery	0.29	0.77	0.60	2.03	9.00	7.00	3.00	9.20
	chirurgien	surgeon	0.50	0.60	0.79	8.45	10.00	8.00	3.00	9.20
	clinique	clinic	0.47	0.44	0.60	11.82	8.00	6.00	2.00	9.10
	guérison	healing	0.75	0.45	0.59	5.20	8.00	5.00	3.00	8.90
	hôpital	hospital	0.32	0.54	0.60	50.41	7.00	4.00	3.00	10.00
	maladie	sickness	0.09	0.70	0.29	49.59	7.00	6.00	3.00	9.00
	médication	medication	0.62	0.44	0.50	0.08	10.00	8.00	4.00	9.00
	nutrition	nutrition	0.88	0.42	0.57	0.34	9.00	8.00	3.00	8.60
	vaccin	vaccinne	0.53	0.57	0.62	4.12	6.00	5.00	2.00	8.80
Mean			0.49	0.55	0.57	14.67	8.22	6.33	2.89	9.09
Standard deviation			0.24	0.13	0.13	20.38	1.39	1.50	0.60	0.39
Immigration	accueil	welcome	0.90	0.56	0.66	16.4	7.00	4.00	2.00	8.00
	arrivant	newcomer	0.62	0.38	0.40	5.07	8.00	5.00	3.00	7.60
	citoyenneté	citizenship	0.79	0.40	0.72	0.34	11.00	7.00	5.00	8.70
	diversité	diversity	0.81	0.47	0.71	3.85	9.00	8.00	4.00	8.20
	frontière	border	0.41	0.38	0.42	26.4	9.00	5.00	2.00	9.10
	migrant	migrant	0.36	0.66	0.51	0.81	8.00	6.00	3.00	9.80
	nationalité	nationality	0.64	0.40	0.77	5.74	11.00	10.00	5.00	8.0
	réfugié	refugee	0.38	0.61	0.33	1.55	7.00	5.00	3.00	8.90
	visa	visa	0.74	0.37	0.66	2.50	4.00	4.00	2.00	9.00
Mean			0.63	0.47	0.58	6.97	8.22	6.00	3.22	8.59
Standard deviation			0.20	0.11	0.16	8.76	2.17	2.00	1.20	0.69
Culture	artiste	artist	0.80	0.44	0.73	28.85	7.00	6.00	2.00	9.90
	bibliothèque	library	625.00	0.14	0.51	40.74	12.00	9.00	4.00	8.10
	cinéma	cinema	0.70	0.54	0.50	78.51	6.00	6.00	3.00	9.10
	langue	language	0.68	0.34	0.70	126.28	6.00	3.00	1.00	8.70
	littérature	literature	0.71	0.25	0.60	36.89	11.00	8.00	4.00	9.40
	musée	museum	0.79	0.21	0.48	28.38	5.00	4.00	2.00	9.50
	musique	music	0.83	0.56	0.61	109.70	7.00	5.00	2.00	9.10
	œuvre	oeuvre	0.65	0.48	0.60	92.23	6.00	3.00	1.00	8.90
	théâtre	theater	0.72	0.73	0.43	64.32	7.00	5.00	2.00	8.90
Mean			70.10	0.41	0.57	67.32	7.44	5.44	2.33	9.07
Standard deviation			208.09	0.19	0.10	36.50	2.40	2.07	1.12	0.51
Neutral words	attelage	hitch	0.51	0.48	0.55	4.05	8.00	5.00	3.00	
	bienveillance	kindness	0.79	0.34	0.72	7.30	13.00	8.00	3.00	
	chantage	blackmail	0.09	0.80	0.48	7.64	8.00	4.00	2.00	
	marque	mark	0.56	0.28	0.55	37.70	6.00	4.00	1.00	
	ménagerie	menagerie	0.57	0.48	0.54	2.09	9.00	7.00	4.00	
	ordre	order	0.60	0.35	0.72	235.34	5.00	4.00	1.00	
	réservation	reservation	0.71	0.50	0.58	0.41	11.00	8.00	4.00	
	technique	technic	0.76	0.41	0.59	14.05	9.00	6.00	2.00	
	trahison	treason	0.83	0.72	0.42	18.51	8.00	5.00	3.00	
Mean			0.57	0.46	0.59	38.57	8.63	5.75	2.50	
Standard deviation			0.22	0.16	0.09	80.39	2.56	1.75	1.20	

### Appendix D. Data Conversion and Normalization

In order to make the data from each measurement comparable between each other, they were placed on the same scale of 0 to 1 (0 being very low salience and 1 being very high salience) and, to do so, many transformations were necessary.

For physiological measurements, the maximum value recorded at each trial is compared to the value before activation. Following the work of Wang et al. (2018), a baseline-correction procedure was applied to both pupillometric and skin conductance signals. For pupil size, a baseline pupil value for each trial was determined by averaging pupil size from 500 ms before the onset of the word presentation. Pupil values were subtracted from this baseline value, and the mean change (from baseline) in an epoch from 500 to 3,000 ms after word onset was used to indicate word-evoked pupil responses. For GSR analyses, baseline (averaging from 1,000 ms before the word appearance) was subtracted from the maximum change between 500 and 3,000 ms after word onset, computed with a log transform (log [GSR]). For each participant, the signal was also standardized in order to take into account the differences in the strengths of the individuals’ physiological reactions. Specifically, we removed the long-term trends in each individual’s sweat rate while preserving the shorter-term fluctuations by fitting a polynomial regression model to the signal and subtracting the fitted curve from the original signal.

In order to analyze the salience of each of the five issues for each respondent, the signals associated with the reactions to neutral words were subtracted from those of the nonneutral words in order to isolate the signals linked to the reaction to the various words. Then, the physiological responses to each issue were calculated by combining the responses to each word by issue category and extracting the mean per ms per issue.

Finally, the data for these two physiological measures were placed on a scale of 0 to 1, by applying a normalization on the aggregated data by issue. Normalization can be expressed as:

$$X_{\mathrm{normalized}}=\frac{\left(X-X_{\mathrm{minimum}}\right)}{\left(X_{\mathrm{maximum}}-X_{\mathrm{minimum}}\right)}$$

where *X* is the measurement for each ms for each measure for each participant. For each respondent, the reaction to stimuli made it possible to identify the issue with the lowest and highest salience score or to remove an issue if it only elicits average reactions.

For self-assessed measurement in the survey, two approaches were used, depending on the type of measure. For selection-type questions, for each question, only one piece of data is captured (respondents must select the most or least important issue, depending on the question; see Appendix B). Thus, for questions relating to the least important issues, 0 is assigned. For responses relating to the most important issues, 1 is assigned. For the scaling-type questions, respondents had to rate the issues on a scale of 0 to 10. The data was therefore simply normalized with the same approach as for the physiological measures. Finally, for both types of measures, the data per respondent was aggregated by issue and summarized by average in order to produce the salience score.

For the objective measurement of response time, a logarithmic transformation was first applied to the objective measurement of response time data, but with data separated for questions on least salient or most salient issues. This transformation allows for correcting skewness in response latency data, although there is no significant difference with or without applying the logarithmic transformation (Christopoulos et al. 2009; Bar-Gill 2007; Leth-Steensen, Elbaz, and Douglas 2000). Data were then normalized by participant with the same process as the other measurement. But, since slower response time is associated with lower salience and shorter response time is associated with higher salience (Simon, 1967; Bishop et al. 2009; Reisenzein and Franikowski 2022; Lavine et al. 1996), the normalized data was reversed. So, normalized data was negatived and then added by 1 to invert the scale of analysis. Thus, data approaching 1 represents a short response time (more salient issue) and data approaching 0 represents a long response time (less salient issue).

### Appendix E. Dispersion of Salience Scores of Each Issue per Participant

All data being normalized, the means by measure for the least and most salient issues for each respondent are at both ends of the scale. Figure E1 presents the mean salience score for both the least and most salient issues for each respondent. The mean of the salience score of the least salient issue approaches 0, although there are variations. For least salient issues, the means for GSR, pupil dilation, and selection measurements are 0, while that of the chronometer is 0.12 and that of the scaling is 0.4. For the most salient issue for each participant, the means are closer to 1 but are more variable: the means for chronometer and selection measurements are 1, the mean for GSR is 0.92, and the means for pupil dilation and scaling measurement are further away, with a value of 0.85. As we see the standard deviation, we can attest that the dispersion of the averages is greater for physiological measurements for the most salient issues.

### Appendix F. Multiple Linear Regression for Each Type of Survey Measurement on Separate Physiological Measures

**Table F1.** Test of the explanatory nature of salience for each type of survey measurement—pupil dilation.

Pupil dilation measurement of issue salience								
	(1)	(2)	(3)	(4)	(5)	(6)	(7)	(8)
Chronometer	0.708	0.717					*−*0.081	*−*0.070
	*p *= 0.000	*p *= 0.000					*p *= 0.044	*p *= 0.083
	(0.046)	(0.046)					(0.040)	(0.040)
Selection			0.692	0.692			0.787	0.792
			*p *= 0.000	*p *= 0.000			*p *= 0.000	*p *= 0.000
			(0.017)	(0.017)			(0.042)	(0.042)
Scaling					1.132	1.143	*−*0.108	*−*0.131
					*p *= 0.000	*p *= 0.000	*p *= 0.138	*p *= 0.079
					(0.048)	(0.048)	(0.072)	(0.074)
Women		*−*0.022		*−*0.022		0.013		*−*0.031
		*p *= 0.638		*p *= 0.512		*p *= 0.786		*p *= 0.357
		(0.046)		(0.033)		(0.047)		(0.033)
No university diploma		*−*0.206		*−*0.121		*−*0.058		*−*0.031
		*p *= 0.021		*p *= 0.058		*p *= 0.514		*p *= 0.058
		(0.087)		(0.063)		(0.088)		(0.060)
Graduate diploma		*−*0.097		*−*0.061		*−*0.044		*−*0.058
		*p *= 0.138		*p *= 0.192		*p *= 0.507		*p *= 0.205
		(0.065)		(0.047)		(0.065)		(0.046)
Between 18 and 24 years old		*−*0.064		*−*0.041		*−*0.116		*−*0.027
		*p *= 0.257		*p *= 0.323		*p *= 0.045		*p *= 0.506
		(0.056)		(0.041)		(0.057)		(0.040)
45 years old and older		0.075		0.032		*−*0.118		0.050
		*p *= 0.372		*p *= 0.586		*p *= 0.162		*p *= 0.402
		(0.083)		(0.059)		(0.084)		(0.059)
Constant	0.185	0.260	0.091	0.146	*−*0.234	*−*0.156	0.133	0.190
	*p *= 0.000	*p *= 0.000	*p *= 0.000	*p *= 0.0000	*p *= 0.003	*p *= 0.522	*p *= 0.000	*p *= 0.000
	(0.027)	(0.055)	(0.018)	(0.040)	(0.035)	(0.060)	(0.031)	(0.046)
N	232	232	232	232	232	232	232	232
Log likelihood	*−*76.647	*−*80.517	105.143	97.945	*−*18.515	*−*24.731	104.674	97.422
AIC	161.295	179.034	*−*202.286	*−*177.891	45.029	67.462	*−*197.348	*−*172.843
BIC	176.368	212.807	*−*187.212	*−*144.118	60.102	101.235	*−*174.776	*−*131.635

*Note:* Linear mixed-effects models. Data from standardized coefficients.

Standard errors presented in parentheses.

**Table F2.** Test of the explanatory nature of salience for each type of survey measurement—galvanic skin response.

Galvanic skin response measurement of issue salience								
	(1)	(2)	(3)	(4)	(5)	(6)	(7)	(8)
Chronometer	0.714	0.720					*−*0.032	*−*0.034
	*p *= 0.000	*p *= 0.000					*p *= 0.624	*p *= 0.485
	(0.047)	(0.048)					(0.049)	(0.049)
Selection			0.675	0.677			0.731	0.729
			*p *= 0.000	*p *= 0.000			*p *= 0.000	*p *= 0.000
			(0.020)	(0.020)			(0.051)	(0.051)
Scaling					1.098	1.116	*−*0.078	*−*0.071
					*p *= 0.000	*p *= 0.000	*p *= 0.509	*p *= 0.428
					(0.052)	(0.052)	(0.088)	(0.090)
Women		*−*0.024		*−*0.021		0.011		*−*0.025
		*p *= 0.616		*p *= 0.601		*p *= 0.814		*p *= 0.531
		(0.048)		(0.040)		(0.046)		(0.040)
No university diploma		*−*0.106		*−*0.008		0.055		*−*0.008
		*p *= 0.239		*p *= 0.912		*p *= 0.527		*p *= 0.917
		(0.090)		(0.075)		(0.086)		(0.076)
Graduate diploma		*−*0.138		*−*0.116		*−*0.093		*−*0.114
		*p *= 0.041		*p *= 0.040		*p *= 0.146		*p *= 0.044
		(0.067)		(0.056)		(0.064)		(0.056)
Between 18 and 24 years old		*−*0.050		*−*0.022		*−*0.100		*−*0.015
		*p *= 0.394		*p *= 0.647		*p *= 0.076		*p *= 0.763
		(0.058)		(0.049)		(0.056)		(0.049)
45 years old and older		*−*0.066		*−*0.109		*−*0.258		*−*0.099
		*p *= 0.446		*p *= 0.126		*p *= 0.002		*p *= 0.171
		(0.086)		(0.071)		(0.082)		(0.072)
Constant	0.137	0.220	0.047	0.107	*−*0.264	*−*0.182	0.076	0.131
	*p *= 0.000	*p *= 0.000	*p *= 0.0004	*p *= 0.0001	*p *= 0.0000	*p *= 0.051	*p *= 0.015	*p *= 0.019
	(0.027)	(0.057)	(0.022)	(0.048)	(0.037)	(0.060)	(0.037)	(0.056)
N	232	232	232	232	232	232	232	232
Log likelihood	*−*85.275	*−*90.955	45.367	40.064	*−*41.020	*−*43.789	42.445	37.099
AIC	178.550	199.910	*−*82.733	*−*62.128	90.041	105.578	*−*72.891	*−*52.199
BIC	193.624	233.683	*−*67.660	*−*28.355	105.114	139.351	*−*50.319	*−*10.992

*Note:* Linear mixed-effects models. Data from standardized coefficients.

Standard errors presented in parentheses.
